# Genetic Characterisation of Closely Related *Lactococcus lactis* Strains Used in Dairy Starter Cultures

**DOI:** 10.3390/ijms27010292

**Published:** 2025-12-27

**Authors:** Yuliya E. Uvarova, Tamara M. Khlebodarova, Asya R. Vasilieva, Aleksandra A. Shipova, Vladimir N. Babenko, Andrey V. Zadorozhny, Nikolay M. Slynko, Natalia V. Bogacheva, Ekaterina Y. Bukatich, Valeriya N. Shlyakhtun, Anton V. Korzhuk, Elena Y. Pavlova, Danil O. Chesnokov, Sergey E. Peltek

**Affiliations:** 1 Laboratory of Molecular Biotechnology, Institute of Cytology and Genetics SB RAS, Lavrentyev Ave. 10, 630090 Novosibirsk, Russia; uvarovaye@bionet.nsc.ru (Y.E.U.); a_ship@bionet.nsc.ru (A.A.S.); bob@bionet.nsc.ru (V.N.B.); bukatich@bionet.nsc.ru (E.Y.B.); shlyakhtun@bionet.nsc.ru (V.N.S.); korshuk_anton@bionet.nsc.ru (A.V.K.); pavlovaey@bionet.nsc.ru (E.Y.P.); chesnokovdo@bionet.nsc.ru (D.O.C.); 2 Kurchatov Genomics Center, Institute of Cytology and Genetics SB RAS, Lavrentyev Ave. 10, 630090 Novosibirsk, Russia

**Keywords:** genetics of lactic acid bacteria, starter cultures, Streptococcaceae, *Lactococcus lactis* subsp. *lactis*, *Lactococcus lactis* subsp. *cremoris*, *Lactococcus lactis* subsp. *diacetilactis*

## Abstract

The complex microbiota of cheese starters plays a key role in determining the structure and flavour of the final product, primarily through their acid-forming capacity, protease activity, and exopolysaccharide synthesis. However, the specific microbial communities underlying the unique qualities of artisanal cheeses remain poorly understood. This study presents the microbiological and molecular genetic characterisation of the microbiome isolated from an artisanal cheese starter in Kosh-Agach, Altai, Russia. Metagenomic analysis of this starter revealed the presence of three bacterial genomes corresponding to those of *Lactococcus lactis*. Pure cultures from this starter were obtained by sequential subculture, and seventeen colonies displaying distinct characteristics on differential media were selected. Genome sequencing was performed for each colony. Bioinformatic analysis based on the *rpoB* gene grouped the isolates into three clusters, each corresponding to a distinct strain of *Lactococcus lactis* subsp. *diacetilactis*. This classification was further confirmed by microbiological and microscopic analyses. A notable finding was that none of the strains produced the characteristic aroma compounds of *L. l.* subsp. *diacetilactis*, namely, diacetyl and CO_2_. The functional properties and metabolic characteristics of this starter consortium are discussed.

## 1. Introduction

Thousands of industrial starters derived from lactic acid bacteria are used worldwide in the production of fermented dairy products. Private farms often use starters derived from unique local bacterial flora, the microbiological structure of which is currently being actively studied [1,2,3,4,5]. When characterising a recently isolated lactic acid bacteria culture, determining its taxonomic position and microbiological characteristics does not fully reflect its industrial potential. Significant intraspecific diversity of lactic acid bacteria is used to produce products with various flavours, aromas, and textural properties characteristic of fermented milk products in different regions. Metagenomic analysis of the microflora of artisanal lactic acid products revealed general trends in the use of specific microorganism species. According to these data, *Lactococcus* predominates in cream and solid products obtained by fermentation of raw milk at room temperature, whereas Lactobacillus predominates in beverages. Furthermore, it has been shown that Streptococcus species predominate in food products made from baked or pasteurised milk and fermented at higher temperatures [1]. The main objective of our study was to investigate the strain composition of the bacterial consortium of the Kraft cheese starter culture from the village of Kosh-Agach, Altai Republic, using metagenomic analysis. Metagenomic analysis of the Kraft starter microflora allows us to determine these characteristics. Genome sequencing of individual strains allows us to identify key gene differences and, based on bioinformatics analysis, construct genetic networks involved in shaping the quality of fermented milk products. Typically, the highly polymorphic species *Lactococcus lactis* is used in cheese starters, which has five subspecies: *L. l. cremoris*, *L. l.* hordniae, *L. l. lactis*, *L. l. lactis* bv. *diacetilactis*, and *L. l. tructae*. Of these, *L. l. lactis*, *L. l. lactis* bv. *L. diacetilactis*, and *L. l. cremoris* exhibit the closest taxonomic relationship [6]. Traditionally, these bacteria have been differentiated using microbiological methods, since microscopic analysis is often insufficient to distinguish between these subspecies of *Lactococcus lactis*. For example, microscopic specimens of *L. l. lactis* and *L. l. diacetilactis* are virtually indistinguishable. Their cells are spherical or oval, 0.5–1.5 μm, and form pairs or short chains [7]. However, they differ from *L. l. cremoris*, which typically forms longer chains. At the same time, the enzymatic properties of *L. l. lactis* and *L. l. diacetilactis* differ significantly. For example, active strains of *L. l. lactis* coagulate milk within 4–6 h, forming a smooth, dense curd and reaching maximum acidity (125 °T) after 5–7 days of development in milk. Strains of this subspecies break down arginine to form ammonia and are used in starters for cottage cheese, sour cream, butter, and low-temperature cheeses. Some strains of *L. lactis* subsp. *lactis* produce the bacteriocin nisin, which exhibits antagonistic activity against most Gram-positive bacteria [8]. *Lactococcus lactis* subsp. *lactis* bv. *diacetilactis*, unlike *L. l. lactis*, is an aromatic streptococcus with relatively weak acid-forming activity. Its strains coagulate milk within 16–18 h, with maximum acidity not exceeding 70–100 °T. The resulting curd often contains gas bubbles (CO_2_) and has a pleasant, characteristic aroma due to the accumulation of diacetyl, as *L. l. Lactococcus lactis* subsp. *cremoris* breaks down lactose and citrates, forming diacetyl, carbon dioxide, and acetoin [9]. These strains are widely used in starters for most fermented dairy products [7]. *Lactococcus lactis* subsp. *cremoris* is characterised as a cream-type streptococcus. Its strains coagulate milk relatively quickly, within 6–8 h, forming a dense curd with a slightly viscous or creamy consistency, which is explained by the ability of *L. l. cremoris* to synthesise polysaccharides. However, the maximum acidity of milk does not exceed 110–115 °T. Unlike *L. l. lactis*, *L. l.* strains of *cremoris* do not metabolise arginine, although some can produce diacetyl depending on the concentration of citric acid in the medium [10]. These strains are used in starters for sour cream, butter, and other fermented dairy products. Some strains of this subspecies can also synthesise the bacteriocin lactococcin [11]. This study examines the microbiological and molecular genetic characteristics of lactic acid bacteria belonging to the species *Lactococcus lactis*, isolated from a farmer’s cheese starter culture, designated as the *lakt1p* sample. The functional properties of the *lakt1p* consortium are also discussed.

## 2. Results and Discussion

### 2.1. Microbiological Characterisation of Pure Cultures Isolated from the lakt1p Starter Culture

**Figure 1:**
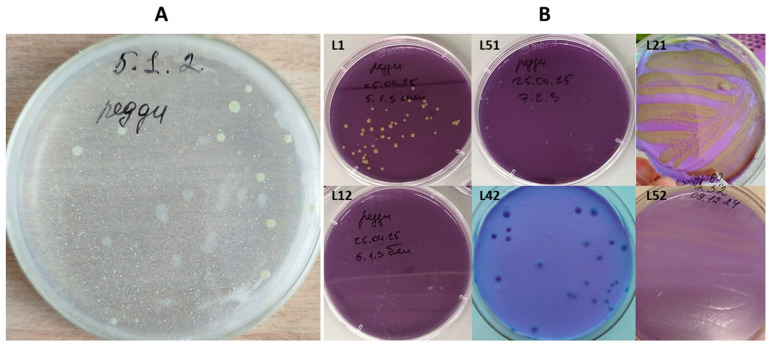
Isolation and differentiation of strains from the *lakt1p* consortium. (**A**) *lakt1p* consortium on Reddy’s differential agar; (**B**) individual colonies after separation of the consortium on the modified Reddy’s agar.

Pure cultures were isolated from the *lakt1p* cheese starter culture sample from a private farm in the Kosh-Agach district of the Altai Republic by sequential subculturing. Seventeen colonies exhibiting distinct morphological characteristics on differential media were selected from these. On Reddy’s differential agar, some colonies exhibited a cream colour, corresponding microbiologically to *L. l.* subsp. *lactis* and *L. l.* subsp. *diacetilactis*, while others were yellow, corresponding to *L. l.* subsp. *cremoris* (Figure 1A).

The yellow colouration observed in *L. l.* subsp. *cremoris* colonies on Reddy’s differential agar is associated with acidification of the medium, as the bromocresol purple indicator acts as a pH-sensitive dye, turning yellow at pH values below 5.2 and purple at pH values above 6.8.

For further differentiation, we used test media based on modified Reddy agar. On these media, *L. lactis* subsp. *diacetilactis* hydrolyses citrate, forming gas bubbles and turning colonies purple (Figure 1B—L51, L12, L42), whereas *L. cremoris* colours colonies yellow (Figure 1B—L1, L21, L52). However, as shown in Figure 1B, none of the cultures tested or any colony in the consortia inoculum (Figure 1A) produced gas bubbles on modified Reddy’s agar. This finding indicates that the cultures under analysis cannot utilise citrate, a trait uncharacteristic of *L. lactis* subsp. *lactis* bv. *diacetilactis*.

Citrate utilisation ability was assessed by performing deep inoculations on a medium containing milk hydrolysate and citrate. However, again, none of the cultures demonstrated this ability.

**Figure 2:**
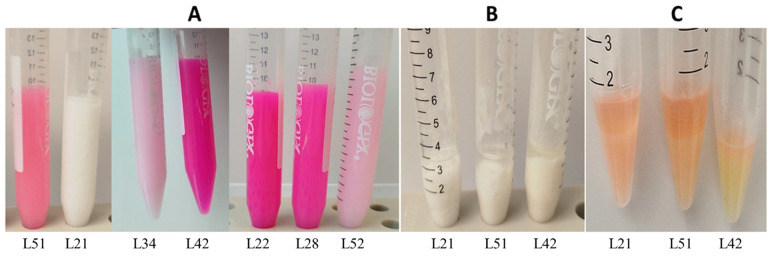
Assessment of microbiological parameters of pure cultures: (**A**) evaluation of culture acidity by titration; (**B**) assessment of carbon dioxide production based on curd characteristics upon heating; (**C**) diacetyl production test after 2 h of cultivation. *X*-axis: sample numbers.

Taxonomic affiliation was determined through a microbiological characterisation of the cultures. This process involved the evaluation of the pure strain activity by measuring the time required for pasteurised milk to curdle after inoculation with 5% of a pure culture and assessing acid-forming activity and curd characteristics. The results showed that the cultures coagulated pasteurised milk within 18–25 h. The acidity of all the samples ranged from 40 °T (pale pink colour, samples L1, L21, L34, and L52) to 80 °T (bright pink colour, samples L42, L22, and L28) (Figure 2A), which corresponds to *L. lactis* subsp. *lactis* bv. *diacetilactis*. However, in all the samples, the curds had a similar texture: they were dense, without gas bubbles and had a consistency of thick sour cream, and upon heating, the curds did not rise but sank to the bottom, indicating the absence of carbon dioxide production (Figure 2B), which is uncharacteristic for *L*. *lactis* subsp. *lactis* bv. *diacetilactis*. Moreover, none of the cultures produced diacetyl, the main aroma compound of *L. l.* subsp. *diacetilactis*. The presence of a pink hue within 10 min is indicative of diacetyl within the starter. In all analysed cultures, the time for the pink colour to appear varied from 52 min to 1.5 h (Figure 2C). Consequently, none of the starter cultures analysed contained diacetyl.

**Figure 3:**
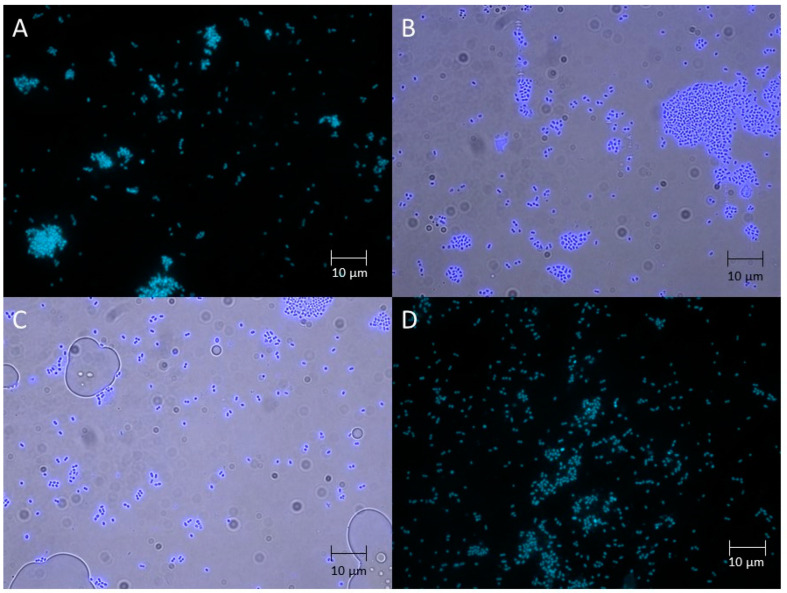
Morphology of the *lakt1p* consortium and pure culture cells with fluorescence (DAPI channel). Scale bar: 10 µm. (**A**) *lakt1p* consortium; (**B**) pure culture sample L21; (**C**) pure culture sample L51; (**D**) pure culture sample L42.

Fluorescent microscopy of the consortium and pure cultures demonstrated that the samples contained spherical cells occurring in groups of 2–4, occasionally forming short chains, approximately 1–1.5 µm in diameter, non-motile and non-spore-forming (Figure 3), which is morphologically consistent with *L. lactis* and *L. lactis* subsp. *diacetilactis*. Most cells were stained with DAPI and exhibited intense fluorescence at 460 nm.

### 2.2. Analysis of the Metagenome of the Microbiological Consortium in Sample lakt1p

Metagenomic analysis of sample *lakt1p* was performed to identify the microbial consortium present in the *lakt1p* cheese starter culture and to facilitate the subsequent isolation of pure cultures. Analysis of the DNA sequencing data obtained from *lakt1p* revealed that the contig sequences belonged to the genus *Lactococcus*. At the same time, it proved challenging to identify the taxonomic classification using 16S rRNA within the metagenomic data set. Table 1 shows the results of mapping the reads from this sample to reference genomes.

In total, 85.9% of reads were mapped to reference genomes of *L. lactis* subsp. *lactis* bv. *diacetilactis*, with 13% assigned to *L. lactis* subsp. *cremoris* and approximately 1% to *L. lactis* subsp. *lactis*.

It should be noted that *L. lactis* subsp. *lactis* and *L. lactis* subsp. *lactis* bv. *diacetilactis* are closely related strains of the same species and therefore have very similar genomes. When using short-read metagenomic sequencing, the low density of genetic differences between these strains makes them difficult to distinguish. Likewise, the contig-binning software cannot distinguish *L. lactis* from *L. lactis* bv. *diacetilactis*. Given the above, only two rather than three distinct genomes were recovered, corresponding to *L. cremoris* subsp. *cremoris* and *L. lactis* subsp. *lactis*. It cannot be excluded that the *lakt1p* sample contains only two strains, *L. cremoris* and *L. lactis* subsp. *Lactis,* which differ from the specific reference genomes chosen (*L. lactis* subsp. *lactis* and *L. lactis* subsp. *lactis* bv. *diacetilactis*).

### 2.3. Bioinformatic Analysis of Sequenced Genomes

Seventeen genomic assemblies were obtained and analysed from individual cultures of the *lakt1p* consortium. Clustering allowed the identification of three clusters based on the *rpoB* gene, one cluster based on the *gyrB* gene, and two clusters according to 16S rRNA. The pairwise ANI comparisons demonstrated 99.9% similarity between assemblies within the same *rpoB* cluster. However, assemblies from different clusters had ANI values ranging from 99.03% to 99.37%. When compared with reference genomes for all the assemblies, the following ANI values were determined: 87.3–87.5% relative to *L. cremoris* subsp. *cremoris* KW2 (GCF_000468955.1); 98–98.3% relative to *L. lactis* subsp. *lactis* strain 14B4 (GCF_003176835.1); and 99.2–99.6% relative to *L. lactis* subsp. *lactis* bv. *diacetilactis* strain S50 (GCF_003627395.2). These findings indicate that the 17 genome assemblies form three *rpoB*-based clusters (Table 2), each representing a distinct strain of *L. lactis* subsp. *lactis* bv. *diacetilactis*.

### 2.4. Mass Spectrometric Analysis of Strains After Separation of the lakt1p Consortium

**Figure 4:**
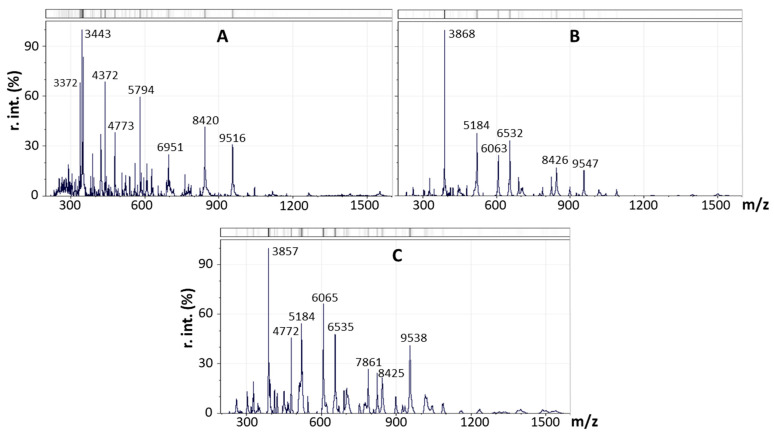
Mass spectra of samples L21 (**A**), L44 (as an example characterising the group of L22/L42/L44/L47/L49) (**B**), and L51 (as an example characterising the group of L12/L28/L41/L45/L46/L51/L53) (**C**). *X*-axis: mass-to-charge ratio (*m*/*z*); *Y*-axis: relative intensity (r/int, %).

Mass spectrometric profiling of 13 cultures from the *lakt1p* consortium enabled their separation into three groups: (1) sample L21; (2) a group of five samples—L22, L42, L44, L47 and L49; (3) a group of seven samples—L12, L28, L41, L45, L46, L51, and L53. The averaged spectra of three experimental repeats for samples L21, L44 and L51 are presented, respectively, in Figure 4A–C.

**Figure 5:**
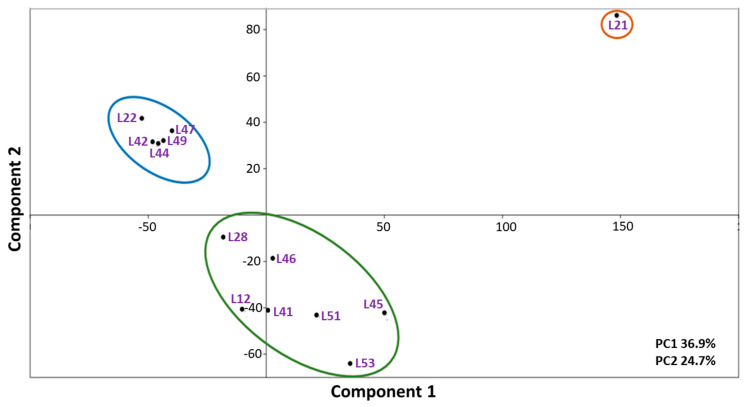
PCA biplot based on all identified *m*/*z* values for each sample.

Samples L22, L42, L44, L47 and L49 (Figure 4B) exhibited similar compositions and intensities of *m*/*z* peaks at 3868, 5184, 6063, 6532, 8426 and 9547. The group comprising L12, L28, L41, L45, L46, L51, and L53 contained several intense peaks shared with the previous group, as well as peaks characteristic of this group alone (*m*/*z* 3857, 4772, 7861, 9538). Sample L21 contained several additional intense *m*/*z* signals, including 3372, 4372, 5794, and 6951. An analysis of the mass spectrograms of the *lakt1p* consortium cultures, based on the *m*/*z* values of all identified mass peaks and their intensities, using principal component analysis, is presented in Figure 5. The analysed samples are clearly divided into three distinct groups.

Further conclusions regarding the similarities and differences between the studied cultures will be made after evaluating the results using other methods (microbiological and genetic).

**Figure 6:**
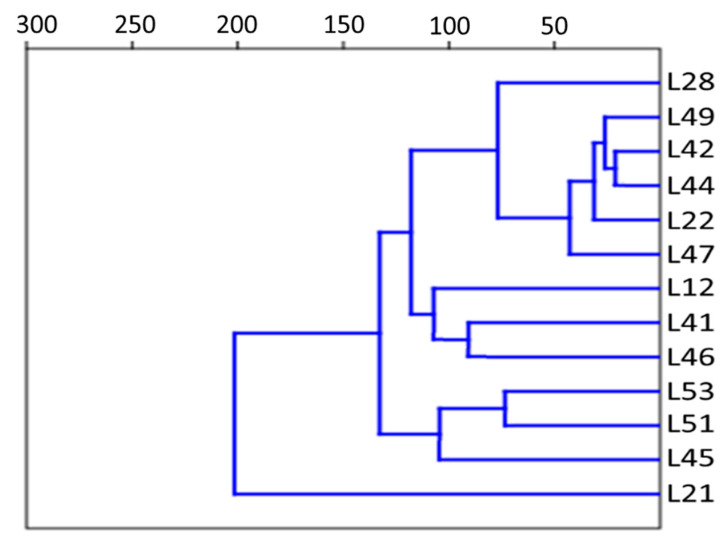
Hierarchical clustering dendrogram based on *m*/*z* values for each sample. *Y*-axis: sample numbers, *X*-axis: dissimilarity index.

Figure 6 presents a dendrogram based on all identified *m*/*z* values, determined using the UPGMA method (Unweighted Pair Group Method with Arithmetic Mean). The analysis of the sample has revealed three clear groupings. The cophenetic correlation coefficient was high (0.9691), indicating that the dendrogram accurately reflects the similarity between the samples.

Hence, mass spectrometric analysis revealed three distinct bacterial groups within the *lakt1p* consortium.

Table 3 summarises the results of the microbiological, genetic, and mass spectrometric analyses of 17 pure cultures derived from the lact1p consortium.

As shown, microbiological analysis on Reddy’s differential medium, along with genomic and mass spectrometric analyses, consistently divided the lact1p pure culture isolates into three clusters. According to phenotypic characteristics based on colony colouration, Cluster 1 was assigned to *L. lactis cremoris*, Cluster 2 to *L. lactis lactis*, and Cluster 3 to *L. lactis diacetilactis*. However, genetic analysis indicated that the three clusters defined by the *rpoB* gene represent distinct strains of *L. lactis diacetilactis*. Microscopic analysis also placed all the strains within *L. lactis diacetilactis*, as the cells were arranged predominantly in pairs (diplococci), virtually without forming chains. Likewise, based on the rate of milk coagulation, the nature of the curd, and final acidity of the samples during milk fermentation, these strains were found to exhibit the traits of *L. lactis diacetilactis*. However, none of the strains, including those identified as *L. lactis diacetilactis* on Reddy differential medium, produced the key aroma-forming compounds characteristic of *L. lactis diacetilactis*, such as diacetyl and CO_2_.

Given the uncertainty regarding the precise taxonomic assignment of the lact1p strains, we sought to identify distinguishing genomic features of the lact1p metagenome. In particular, it is worth performing a comparison with the reference genome of *Lactococcus lactis* subsp. *lactis* and with a synthetic metagenome composed of reference sequences from *L. lactis cremoris*, *L. lactis lactis* and *L. lactis diacetilactis*. Special attention should be paid to genes involved in the synthesis of enzymes, pheromones, exopolysaccharides and other compounds responsible for the flavour, aroma and textural properties of the final product in the *lakt1p* consortium strains.

Furthermore, the division of the *lakt1p* consortium into three clusters, based on genomic, proteomic, and phenotypic characteristics observed in differential media, necessitates supplementary comparative bioinformatic analyses of genomic traits among the strains within these clusters. The following sections of the article address these questions in detail.

### 2.5. Comparison of Exopolysaccharide Synthesis Genes in the Metagenome of the lakt1p Starter Culture and the Synthetic Metagenome

As noted above, *L. lactis* subsp. *cremoris* is characterised as a “creamy” streptococcus, owing to its higher capacity for exopolysaccharide (EPS) synthesis and secretion compared with other *L. lactis* subspecies. Microbiological tests of individual colonies from the *lakt1p* starter on Reddy’s differential agar exhibited a yellow colouration, which, according to microbiological characteristics, is typical of *L. l. cremoris* (Figure 1A), suggesting that this subspecies may indeed be present in the *lakt1p* culture. This assumption is also consistent with the metagenomic analysis of *lakt1p*.

Exopolysaccharide synthesis in lactic acid bacteria is facilitated by an extensive pool of glycosyltransferases with a broad spectrum of sugar and linkage specificities. Exopolysaccharides secreted by lactic acid bacteria can be directly applied to achieve the desired viscosity, texture, and mouthfeel in food production, particularly due to their water-binding capacity, gel formation, flow behaviour and overall rheological performance of the final product [12]. Exopolysaccharides exhibit great structural diversity, and some provide specific biological activity and physiological functions, such as antioxidant activity [13], antimicrobial effects [14], and immunostimulatory effects [15], among others.

We compared the set of glycosyltransferases identified in the metagenome of the *lakt1p* sample and found that, when compared with the genome of *L. lactis* subsp. *lactis*, the glycosyltransferase repertoire was identical. However, comparison with a synthetic metagenome composed of reference sequences from *L. l. cremoris*, *L. l. lactis* and *L. l. diacetilactis* revealed that the *lakt1p* sample contains genes encoding glucosyltransferase, oligosaccharide 4-α-D-glucosyltransferase and poly(ribitol-phosphate) β-glucosyltransferase, which are absent from the annotated synthetic metagenome. This indicates certain genetic differences between the strains in the *lakt1p* consortium and the reference genomes of *L. lactis cremoris* and *L. lactis diacetilactis*.

### 2.6. Analysis of the Peptidase Spectrum in the lakt1p Metagenome and in the Isolated Cultures

Given the strong correlation between high acidification capacity of starter strains and the proteolytic activity of their proteinases [16,17], consideration should be given to the fact that lactic acid bacterial strains may differ substantially in both the spectrum and activity of their proteinases. Consequently, we analysed the peptidase repertoire in the *lakt1p* sample and in the reference genomes. The results are summarised in Table 4 and Table 5.

As shown in Table 4, the *lakt1p* metagenome shares 11 peptidases with the *L. l. lactis* reference genome. It also contains five peptidases that are not annotated in the reference genome, whereas the reference genome encodes eight peptidases that were not annotated in the *lakt1p* metagenome. Table 5 shows that the *lakt1p* metagenome shares 14 peptidases with the synthetic metagenome. The *lakt1p* sample also contains four peptidases absent from the annotations of *L. l. cremoris*, *L. l. lactis* and *L. l. diacetilactis*, while three reference species collectively possess ten peptidases that were not annotated in the *lakt1p* metagenome.

Genomic DNA analysis of 17 individual strains from the *lakt1p* consortium revealed that all 17 genomes encoded peptidases: Neutral endopeptidase (*pepO_*1, *pepO_*2), Dipeptidase A (*pepDA*_1, *pepDA*_2), Beta-Ala-Xaa dipeptidase (*pepV*), and Xaa-Pro dipeptidase (*pep*Q). Notably, only genomes from the third cluster (based on genomic analysis) encoded the peptidase PepF1 (EC 3.4.24, oligoendopeptidase F), which appears to be plasmid-borne. Differences observed between the isolated strains may be explained by plasmid loss in some strains, which could underlie the morphological variation.

Pheromones, exopolysaccharides and other compounds contribute to the flavour, aroma, and textural properties of the final fermented product. The presence of genes encoding these traits in the *lakt1p* lactococcal strains is summarised in Table 6. Shown are clusters identified through genome analysis.

The genomic analysis presented in Table 6 demonstrates that the individual strains of the *lakt1p* consortium have genes for most enzymes responsible for lactose utilisation, synthesis of exopolysaccharide precursors with diverse sugars, including glucose, galactose, and rhamnose. Only a single bacteriocin-encoding gene was identified: the *lcnB* gene for lactococcin B, which is present in clusters 2 and 3 and absent from cluster 1 strains. It has been reported that lactococcin B can be produced by some strains of *Lactococcus lactis* subsp. *lactis* [18] as well as by *L. lactis* subsp. *cremoris* [19], and thus does not allow subspecies identity of the *lakt1p* clusters to be determined unambiguously.

The exopolysaccharide synthesis gene cluster contains only the *epsL* gene. In *Bacillus subtilis*, this gene produces a phosphoglycosyltransferase. This enzyme initiates exopolysaccharide unit synthesis, utilising UDP-di-N-acetylbacillosamine as a phosphosaccharide donor [20]. These findings suggest that a larger EPS gene cluster may be located on plasmids.

### 2.7. Bioinformatic Analysis of Gene Interaction Networks in Strains L21, L42 and L51 from the lakt1p Consortium

As demonstrated above, genomic analysis of the *rpoB* gene (Table 2) and mass spectrometry (Figure 6) reveal that strains L21, L42, and L51, obtained from the *lakt1p* consortium, belong to different clusters. Furthermore, on Reddy’s differential agar, the colonies of these strains exhibited differential staining, indicative of their belonging to distinct subspecies of *Lactococcus lactis*. According to differential colouration, L21 corresponds to cluster 1 (*L. lactis cremoris*), L51 to cluster 2 (*L. lactis lactis*) and L42 to cluster 3 (*L. lactis diacetilactis*) (Table 3). These strains were therefore selected as representative genomes for each cluster.

**Figure 7:**
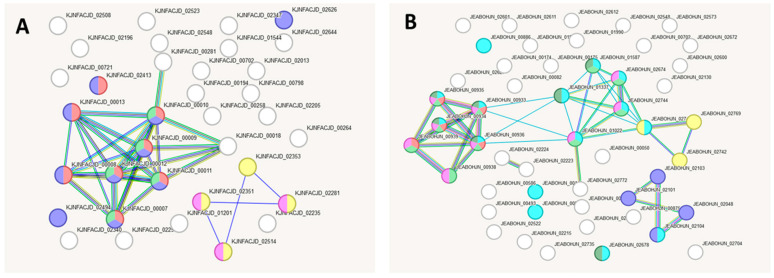
Reconstruction of the gene interaction network based on genes unique to strains L21 and L42 when compared with each other. (**A**) L21 vs. L42 version; (**B**) L42 vs. L21 version.

To determine the gene groups responsible for the aforementioned metabolic characteristics in these strains, we performed a comparative genomic analysis and compiled unique gene lists for each strain through pairwise comparisons (Appendix A, Table A1, Table A2 and Table A3). Based on the data obtained, a pairwise comparison of the gene networks of strains L21, L42, and L52 was conducted using the string-db resource. Below, three pairs of gene interaction networks are presented: L21 vs. L42, L42 vs. L51, and L21 vs. L51, each characterised by specific Gene Ontology (GO) categories. For each comparison, the most reliable GO category (based on the number of genes involved) is reported. Figure 7 shows the reconstructed interaction networks based on genes differentiating strains L21 and L42. One network includes 37 genes present in L21 but absent from L42 (Table A1; Figure 7A), while the other comprises 44 genes present in L42 but absent from L21 (Table A1; Figure 7B).

Table 7 and Table 8 provide the data on the gene networks specific to strains L21 and L42, with a focus on their GO characteristics and how they differ from each other.

**Figure 8:**
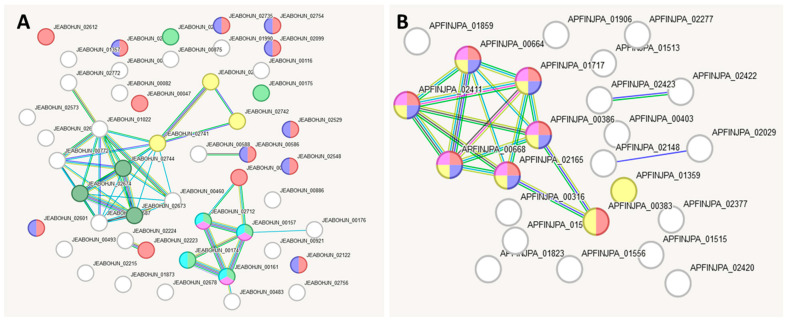
Reconstruction of the gene interaction network based on genes unique to strains L42 and L51 when compared with each other. (**A**) L42 vs. L51 version; (**B**) L51 vs. L42 version.

The protein-coding genes present in strain L42 but absent in L21 are involved in membrane transport processes (Figure 8B, Table 8), including the transport of peptides and metal ions. This indicates a potentially greater capacity of strain L42 to maintain intracellular homeostasis and to utilise protein derivatives. At the same time, the gene network of strain L21 compared with L42 (Table 7) contains GO categories associated with *oxo-acid lyase activity*, which suggests a potential ability of L21 to metabolise oxo-acids, including citric acid.

Figure 8 shows the reconstructed gene interaction networks based on genes distinguishing strains L42 and L51 from each other. One network comprises 44 genes present in strain L42 but absent in L51 (Table A2; Figure 8A), while the other comprises 27 genes present in L51 but absent in L42 (Table A2; Figure 8B).

According to the data presented in Figure 8 and Table 9 and Table 10, the protein-coding genes present in strain L42 but absent in strain L51 are involved in the biosynthesis of polysaccharides, including pathways using galactose as a precursor. In turn, the protein-coding genes found in strain L51 but absent in L42 are implicated in the metabolism of sugars—including galactose—and polysaccharides. These findings suggest that the differences between strains L42 and L51 are minor and are potentially associated with the involvement of the products of their unique genes in the same polysaccharide biosynthesis processes, albeit at different stages of their implementation.

**Figure 9:**
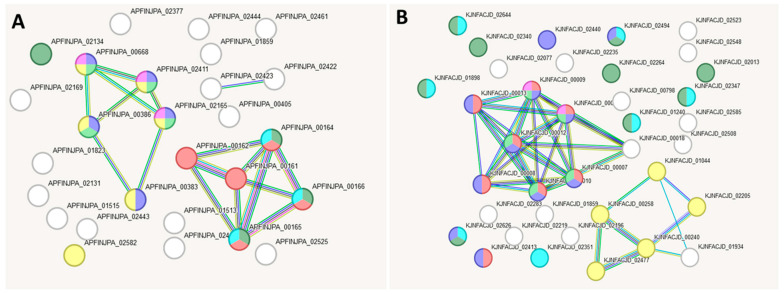
Reconstruction of the gene interaction networks based on genes unique to strains L51 and L21 when compared with each other. (**A**) L51 vs. L21 version; (**B**) L21 vs. L51 version.

Figure 9 shows the reconstructed gene interaction networks based on genes distinguishing strains L51 and L21 from each other, comprising 28 genes present in strain L51 but absent in L21 (Table A3; Figure 9A) and 37 genes present in strain L21 but absent in L51 (Table A3; Figure 9B).

According to the data provided in Figure 9 and Table 11, the protein-coding genes present in strain L51 but absent in strain L21 are involved in transport processes, signal transduction pathways and carbohydrate metabolism. By contrast, the principal distinction between strain L21 and strain L51 (see Table 12) is the presence in L21 of a citrate utilisation gene cluster, which enables this strain to metabolise citrate.

Thus, the comparative analysis indicates that the sets of protein-coding genes underlying the differences between strains L51 and L42 exhibit only minor variation in metabolic potential, primarily in the repertoire of enzymes involved in carbohydrate metabolism and polysaccharide biosynthesis. In contrast, the genome of strain L21 contains genes encoding the key enzyme of citrate metabolism, a feature characteristic of the subspecies *L. lactis diacetilactis* [9], although certain strains of *L. lactis cremoris* may also possess this capability [10].

Since none of the lact1p strains, including L21, can synthesise diacetyl or CO_2_ from citrate, we hypothesise that strain L21 may have lost this ability. The reason for this loss remains unclear. When considering potential causes of this loss, it is important to acknowledge that the activity of the citrate lyase complex depends on the activity of citrate permease, a transport protein required for citrate uptake from the medium. Notably, the gene encoding this citrate-specific transporter in *L. lactis diacetilactis* is plasmid-borne [9]. Since the current analysis was limited to chromosomal genomic sequences, it proved impossible to determine if strain L21 possesses the plasmid-encoded *citP* gene, which codes for citrate permease.

## 3. Materials and Methods

In this study, we examined a cow’s milk cheese starter culture from a farm in the village of Kosh-Agach (Altai Republic). The starter culture (named *lakt1p*) was aseptically collected into a sterile container. Metagenomic DNA was extracted from the starter culture to analyse the taxonomic affiliation of the cultures in the consortium. The *lakt1p* starter culture was also separated into individual cultures by sequential subculture on milk agar, MRS and M17 media (Condalab, Madrid, Spain), and Reddy’s differential agar at 30 °C. Seventeen individual colonies were selected for further analysis.

### 3.1. Microbiological Methods

Microbiological characterisation of the pure cultures was performed by inoculating them onto test media. The Reddy’s differential agar medium was prepared with the following components (%): Tryptone, 0.5; CaCO_3_, 0.3; carboxymethyl cellulose (CMC), 0.6; K_2_HPO_4_, 0.1; yeast extract, 0.5; l-arginine hydrochloride, 0.5; agar-agar, 1.5, 5 mL sterile skim milk; and 0.1% bromocresol purple indicator solution, 2 mL (pH 6.3). On this medium, *L. lactis* subsp. *cremoris*, which does not hydrolyse arginine, forms yellow colonies, while *L. lactis* subsp. *lactis* and *L. lactis* subsp. *diacetilactis* form white colonies.

A modified Reddy’s differential agar was also used, containing (g/L): CMC, 5; sodium citrate, 10; peptone, 7.5; yeast extract, 7.5; meat extract, 5; lactose, 1.5; L-Arginine hydrochloride, 1.5; bromocresol purple, 0.002; and agar-agar, 15 (pH 6.0).

Citrate utilisation was assessed using a medium comprising milk hydrolysate, 2.5% yeast autolysate, 2.0% agar-agar, 2 mL of 0.1% bromcresol purple indicator solution, and 1% sodium citrate. After 18–24 h of cultivation at 30 °C in this enrichment medium, the bacterial suspension was plated using the deep-pour method onto the solidified medium of the same composition. Colonies of *L. l. diacetilactis* were identified by the gas bubble formation resulting from citrate metabolism.

The acidification activity of pure strains was evaluated by measuring the time required for pasteurised milk to coagulate after inoculation with 5% of the pure culture starter. Titratable acidity was measured by adding 10 mL of the starter culture, 20 mL of distilled water, and three drops of phenolphthalein to a 100 mL flask, then titrating with a 0.1 N NaOH solution until a pink colour appeared. The volume of NaOH (in mL) multiplied by 10 corresponds to the acidity of the sample, expressed in Turner degrees (°T).

Carbon dioxide and four-carbon compounds are metabolic products of the aroma-forming bacterium *L. l. diacetilactis*. Carbon dioxide production was quantified by thoroughly homogenising the starter culture and pipetting 20 cm^3^ into a tube (15 mm diameter). The initial level of the culture was marked, and the tube was placed in a water bath containing cold water. The water was then heated to 90 °C, and the elevation of the curd was recorded without removing the tube. In the presence of carbon dioxide, the curd becomes spongy and rises 0.6–3 cm or more above the whey. In the absence of carbon dioxide, the curd typically does not rise and may even sink to the bottom.

Determination of diacetyl and acetoin was performed by mixing the starter culture and filtering it through a paper filter. To 200 µL of the filtrate, 200 µL of a 40% KOH solution was added, followed by thorough mixing. The time required for the development of a pink colour was recorded. If the colour appeared within 10 min, the presence of diacetyl in the starter culture was inferred.

### 3.2. Sample Morphology Analysis by Fluorescence Microscopy

Cells grown on a solid medium were used for microscopy. For each sample, at least three preparations were examined, with a minimum of 10 fields of view counted in each preparation. Fixed preparations were prepared from each sample and subsequently stained with DAPI in PBS buffer. Morphological analysis was performed using Karl Zeiss (Jena, Germany) microscopes: Axio Imager.M2 and Axio Imager.A2 (microscopic analysis was carried out at the Multiple-access Center for Microscopy of Biological Objects (Institute of Cytology and Genetics, Novosibirsk, Russia)) and Axio Scope.1 (Laboratory of Molecular Biotechnology, IC&G SB RAS, Akademgorodok, Russia).

### 3.3. DNA Extraction, Library Preparation, Sequencing

Genomic DNA was extracted from pure microbial cultures. The cells were resuspended in TE buffer (pH 8), and genomic DNA was isolated using the phenol–chloroform method. Plasmid DNA was not sequenced separately. The quality of the extracted DNA was assessed spectrophotometrically (Epoch, BioTek Instruments, Winooski, VT, USA) and using a Qubit fluorometer (Quant-iT™ dsDNA Assay Kit, High Sensitivity (HS), Thermo Fisher, Waltham, MA, USA). Library preparation and sequencing were performed at the Sequencing Centre of IC&G SB RAS using an Illumina MiSeq platform (version 4.1.0).

### 3.4. Bioinformatic Analysis of Sequencing Data

Quality check of metagenomic and individual strain sequencing raw reads was performed using FastQC (version 0.11.8), followed by trimming with Cutadapt (version 1.18). Metagenomic reads were aligned to the following reference genomes available in the NCBI database: *Lactococcus cremoris* subsp. *cremoris* KW2 (GCF_000468955.1), *Lactococcus lactis* subsp. *lactis* strain 14B4 (GCF_003176835.1), and *Lactococcus lactis* subsp. *lactis* bv. *diacetilactis* strain S50 (GCF_003627395.2), using Bowtie2 (version 2.3.5). Genome and metagenome assemblies were generated using SPAdes (version 4.0.0). The assembled metagenomic contigs were classified using MetaWRAP (version 1.2.2, classify_bins option), and the proportion of contigs assigned to each taxonomic unit was calculated. Genome and metagenome annotation was performed using PROKKA (version 1.14.6). Annotated genomes of individual samples were screened for the *16S*, *rpoB*, and *gyrB* genes to determine the taxonomic identity of the strains. Clustering of the identified sequences was performed using CD-HIT (version 4.7). Average nucleotide identity (ANI) values for the assemblies were calculated using fastANI (version 1.34). A synthetic metagenome consisting of *L. l. cremoris*, *L. l. diacetilactis*, and *L. l. lactis* was constructed by combining the sequences of the corresponding reference genomes (GCF_000468955.1, GCF_003627395.2, and GCF_003176835.1) into a single file.

Seventeen genome assemblies from individual cultures of the *lakt1p* consortium were obtained and analysed. Sequence similarity clustering was performed using rpoB, and a genome from each cluster was selected for further bioinformatics analysis (L51, L42, L21).

### 3.5. Mass Spectrometric Analysis of Strain Proteomes

Proteomic analysis was performed using matrix-assisted laser desorption/ionisation time-of-flight mass spectrometry (MALDI-TOF MS).

### 3.6. Preparation of Bacterial Cell Lysates for MALDI-TOF MS Measurement

For each colony analysed, approximately 50 µL of culture was collected. The cells were resuspended in 300 µL of deionised water, followed by the addition of 900 µL of 96% ethanol for inactivation. The cells were pelleted by centrifugation for 2 min at 15,600× *g*. The supernatant was removed, and the pellet was dried for 5 min in an Eppendorf vacuum concentrator. Bacterial cell walls were disrupted by adding 50 µL of 70% formic acid. Proteins were extracted with 50 µL of acetonitrile. The mixture was centrifuged for 2 min at 15,600× *g*, and the supernatant was transferred to a clean tube for mass spectrometric analysis.

### 3.7. Conditions for MALDI-TOF MS Measurement

For mass spectrometric analysis, 1 µL of the protein extract was applied to a stainless-steel plate and allowed to dry at room temperature. Subsequently, 1 µL of matrix solution (6 mg/mL α-cyano-4-hydroxycinnamic acid in acetonitrile/water/trifluoroacetic acid, 50:47.5:2.5, *v*/*v*) was overlaid onto the sample.

Measurements were performed using an Ultraflex III MALDI-TOF/TOF mass spectrometer (Bruker Daltonics, Billerica, MA, USA). Spectra were acquired in linear positive mode with a laser frequency of 200 Hz over a mass range of 2000–20,000 Da. The accelerating voltage was set to 20 kV, IS2 voltage to 18.66 kV, and lens voltage to 6 kV, with extraction delay disabled. The obtained spectra represent the dependence of the mass of the sample proteins (*m*/*z*) and their intensity.

For each colony, the spectra were obtained by summing 2000 laser shots (10 × 200 shots from different target spot positions). External calibration was performed using the accurate mass values of known *Escherichia coli* proteins: RL36—4365.3 Da, RS22—5096.8 Da, RL34—5381.4 Da, RL32—6315.0 Da, RL29—7274.5 Da, and RS19—10,300.1 Da.

The acquired spectra were exported to mMass. A baseline subtraction procedure was applied to all spectra using the MALDI-TOF Proteins 5–20 kDa preset with default settings. Peak picking was performed using the following parameters: signal-to-noise threshold—2.5, absolute intensity threshold—0, relative intensity threshold—1%, and picking height—100%.

### 3.8. Statistical Analysis of Mass Spectra

Multivariate statistical analysis was performed using Past 5 software, version 5.0 [21]. Hierarchical cluster analysis was used to visualise the differences between the strains analysed. Multivariate approaches, particularly principal component analysis (PCA), were applied for a more comprehensive and in-depth evaluation. All *m*/*z* values and their intensities were used as variables for the principal component analysis (PCA).

### 3.9. Bioinformatic Analysis: Gene Network Construction

A comparative analysis of gene networks constructed from genes unique to each strain was performed to identify specific metabolic features of strains L21, L42, and L51 isolated from the lact1p sample. The STRING database application (https://string-db.org/, accessed on 23 December 2025) [22] was used to identify associated gene networks.

For each clone (L51, L42, L21), genome protein annotations were uploaded to STRING using the corresponding identifiers: L51—STRG0A09PVY, L42—STRG0A14NDF, and L21—STRG0A47PZJ.

The string-db.org application was configured with the following parameters: (1) meaning of network edges—evidence; (2) types of evidence used—text mining, experiments, databases, co-expression, neighbourhood, gene fusion, co-occurrence; (3) minimum required interaction score—minimum confidence 0.41; and (4) max number of interactions to show—no more than 10 interactions.

When describing the strain comparisons, the most reliable Gene Ontology (GO) categories (based on the number of genes represented) were selected.

## 4. Conclusions

This study characterised the microbiological and molecular genetic properties of lactic acid bacteria belonging to the species *Lactococcus lactis* isolated from a farm cheese starter culture (sample *lakt1p*). Pure cultures were obtained from this sample by sequential subculturing, and 17 colonies exhibiting different characteristics on differential media were selected. Genetic analysis based on the *rpoB* gene grouped these colonies into three clusters, each corresponding to a distinct strain of *L. lactis* subsp. *diacetilactis*. Microbiological and microscopic analyses further confirmed that all the strains could be assigned to *L. lactis* subsp. *diacetilactis*. However, none of the studied strains produced the characteristic aroma compounds of *L. l. diacetilactis*, such as diacetyl and CO_2_. Phenotypically, based on colony colouration on Reddy’s differential media, the strains were also divided into three clusters.

The functional characteristics of the *lakt1p* consortium strains were further explored using the STRING database. Pairwise comparisons of gene sets unique to strains L21, L42, and L52, which were assigned to different clusters based on genomic, proteomic, and phenotypic features, were performed. Gene network analysis revealed that the unique protein-coding genes distinguishing strains L51 and L42 conferred only minor differences in metabolic potential, primarily in the repertoire of enzymes involved in sugar metabolism and polysaccharide biosynthesis. In contrast, the gene network of unique protein-coding genes in strain L21 included a cluster of genes from the citrate lyase cluster, a feature characteristic of *L. lactis* subsp. *diacetilactis* [9]. Nevertheless, the inability of the *lakt1p* strains, including L21, to synthesise diacetyl and CO_2_ due to citrate utilisation suggests that at least L21 may have once possessed this ability but subsequently lost it. This loss could be associated with the absence of a functional citrate permease, which may be encoded on a plasmid.

## Figures and Tables

**Table 1:** Proportion of reads from the *lakt1p* sample mapped to reference genomes.

Proportion of Reads in the Metagenome, %	Reference Genome Used for Read Alignment
**85.9**	***Lactococcus lactis*** **subsp. *diacetilactis***
25	NZ CP061325.1 *Lactococcus lactis* subsp. *lactis* bv. *diacetilactis* strain S50 plasmid pS6, complete sequence
20	NZ CP061326.1 *Lactococcus lactis* subsp. *lactis* bv. *diacetilactis* strain S50 plasmid pS74, complete sequence
15	NZ CP061327.1 *Lactococcus lactis* subsp. *lactis* bv. *diacetilactis* strain S50 plasmid pS7a, complete sequence
8	NZ CP061328.1 *Lactococcus lactis* subsp. *lactis* bv. *diacetilactis* strain S50 plasmid pS7b, complete sequence
6.7	NZ CP061322.1 *Lactococcus lactis* subsp. *lactis* bv. *diacetilactis* strain S50 chromosome, complete genome
5.6	NZ CP061323.1 *Lactococcus lactis* subsp. *lactis* bv. *diacetilactis* strain S50 plasmid pS127, complete sequence
5.6	NZ CP061324.1 *Lactococcus lactis* subsp. *lactis* bv. *diacetilactis* strain S50 plasmid pS19, complete sequence
**13**	***Lactococcus lactis*** **subsp. *cremoris***
13	NC 022369.1 Lactococcus *cremoris* subsp. *cremoris* KW2, complete sequence
**1.0061**	***Lactococcus lactis*** **subsp. *lactis***
1	NZ CP028160.1 *Lactococcus lactis* subsp. *lactis* strain 14B4 chromosome, complete genome
0.0061	NZ CP028161.1 *Lactococcus lactis* subsp. *lactis* strain 14B4 plasmid p14B4, complete sequence

**Table 2:** *rpoB*-based clusters of *lakt1p* consortium strains.

Cluster Number	Strain Name
Cluster 1	L1, L21, L34, L52
Cluster 2	L12, L41, L45, L46, L47, L49, L51, L53
Cluster 3	L22, L28, L29, L42, L44

**Table 3:** Summary of microbiological, genetic and mass spectrometric analyses of pure cultures *.

Strain Number	Cluster Number, Genomic Analysis	Cluster Number, Mass Spectrometry Analysis	Colour on Reddy’s Agar	Coagulation Time, Hours	Diacetyl Test Time, Min	Sample Acidity, °T
L1	1	–	yellow	16	58	40
L21	1	1	yellow	17	63	40
L34	1	–	yellow	18	52	40
L52	1	–	yellow	18	72	40
L12	2	2	white	19	61	65
L41	2	2	white	20	67	65
L45	2	2	white	20	80	65
L46	2	2	white	20	87	65
L47	2	3	white	18	93	65
L49	2	3	white	17	85	65
L51	2	2	white	19	88	65
L53	2	2	white	18	84	65
L22	3	3	purple	17	76	80
L28	3	2	purple	18	84	80
L29	3	–	purple	19	73	75
L42	3	3	purple	18	70	80
L44	3	3	purple	20	64	75

All the cultures lacked citrate-hydrolysing ability and did not produce CO_2_. All of them formed dense, sour-cream-like coagula during milk fermentation.

**Table 4:** Spectrum of peptidases in the *lakt1p* sample obtained by comparing the metagenome with the genome of *Lactococcus lactis* subsp. *lactis*.

N	Genes Encoding Peptidases		
	Unique to the *lakt1p* Metagenome	Unique to the *L. l.* subsp. *lactis* Genome (GCF_003176835.1)	Common to the Reference Genome *L. l.* subsp. *lactis* (GCF_003176835.1) and the *lakt1p* Sample
1	Aminopeptidase YpdF	Beta-Ala-Xaa dipeptidase	Aminopeptidase C
2	D-alanyl-D-alanine carboxypeptidase DacA	D-alanyl-D-alanine carboxypeptidase DacA precursor	Aminopeptidase N
3	Putative D-alanyl-D-alanine carboxypeptidase	Gamma-D-glutamyl-L-lysine endopeptidase	D-alanyl-D-alanine carboxypeptidase
4	Signal peptidase I U	Glutamyl aminopeptidase	Dipeptidase A
5	Pyrrolidone-carboxylate peptidase	Murein tetrapeptide carboxypeptidase	Lipoprotein signal peptidase
6		Peptidoglycan DL-endopeptidase CwlO precursor	Methionine aminopeptidase 1
7		putative murein peptide carboxypeptidase	Neutral endopeptidase
8		putative peptidase	Oligoendopeptidase F, plasmid
9			Peptidase T
10			Signal peptidase I T
11			Xaa-Pro dipeptidase

**Table 5:** Spectrum of peptidases in the *lakt1p* sample obtained by comparing the *lakt1p* sample with a synthetic metagenome composed of reference genome sequences of *L. lactis* subsp. *cremoris*, *L. lactis* subsp. *diacetilactis*, and *L. lactis* subsp. *lactis*.

N	Genes Encoding Peptidases		
	Unique to the *lakt1p* Metagenome	Unique to the Synthetic Metagenome (from Reference Genomes of *L. l.* subsp. *cremoris*, *L. l.* subsp. *diacetilactis*, *L. l.* subsp. *lactis*)	Common to the Synthetic Metagenome and the *lakt1p* Sample
1	Aminopeptidase YpdF	D-alanyl-D-alanine carboxypeptidase DacA precursor	Aminopeptidase C
2	D-alanyl-D-alanine carboxypeptidase DacA	D-gamma-glutamyl-meso-diaminopimelic acid endopeptidase CwlS precursor	Aminopeptidase N
3	Putative D-alanyl-D-alanine carboxypeptidase	Gamma-D-glutamyl-L-lysine endopeptidase	Beta-Ala-Xaa dipeptidase
4	Pyrrolidone-carboxylate peptidase	Glutamyl endopeptidase precursor	D-alanyl-D-alanine carboxypeptidase
5		Murein DD-endopeptidase MepM	Dipeptidase A
6		Murein tetrapeptide carboxypeptidase	Glutamyl aminopeptidase
7		Peptidoglycan DL-endopeptidase CwlO precursor	Lipoprotein signal peptidase
8		putative endopeptidase precursor	Methionine aminopeptidase 1
9		putative murein peptide carboxypeptidase	Neutral endopeptidase
10		putative peptidase	Oligoendopeptidase F, plasmid
11			Peptidase T
12			Signal peptidase I T
13			Signal peptidase I U
14			Xaa-Pro dipeptidase

**Table 6:** Genes responsible for the synthesis of pheromones, exopolysaccharides, and other compounds determining the flavour, aroma and textural properties of the final product in strains of the *lakt1p* consortium.

Cluster1	Cluster 2	Cluster 3	EC Number	Gene Name	Process
	*lcnB*	*lcnB*		Bacteriocin lactococcin B	Antimicrobial peptide (bacteriocin) synthesis
*epsL*	*epsL*	*epsL*	2.-.-.-	putative sugar transferase EpsL	Exopolysaccharide biosynthesis
*pgmB*	*pgmB*	*pgmB*	5.4.2.6	Beta-phosphoglucomutase	Biosynthesis of EPS precursors containing glucose and galactose
*gtaB*	*gtaB*	*gtaB*	2.7.7.9	UTP-glucose-1-phosphate uridylyltransferase	
*galK_1*	*galK_1*	*galK_1*	2.7.1.6	Galactokinase	
*galK_2*	*galK_2*	*galK_2*	2.7.1.6		
*galT*	*galT*	*galT*	2.7.7.12	Galactose-1-phosphate uridylyltransferase	
*mro*	*mro*	*mro*	5.1.3.3	Aldose 1-epimerase (mutarotase)	
	*galE*		5.1.3.2	UDP-glucose 4-epimerase	
*rfbA*	*rfbA*	*rfbA*	2.7.7.24	Glucose-1-phosphate thymidylyltransferase 1	Biosynthesis of EPS precursors containing rhamnose
*rfbC_1*	*rfbC*	*rfbC_1*	5.1.3.-	Protein RmlC	
*rfbC_2*		*rfbC_2*	5.1.3.13		
	*rfbB*		4.2.1.46	dTDP-glucose 4,6-dehydratase	
*lacS*	*lacS*	*lacS*		Lactose permease	Transport of lactose and galactose
*lacZ*	*lacZ*	*lacZ_1*	3.2.1.23	Beta-galactosidase	Hydrolysis of lactose into glucose and galactose
		*lacZ_2*	3.2.1.23		
*mnaA*	*mnaA*	*mnaA*	5.1.3.14	UDP-N-acetylglucosamine 2-epimerase	Teichoic acid biosynthesis
*ywqD*	*ywqD*	*ywqD*	2.7.10.2	Tyrosine-protein kinase YwqD	Capsular polysaccharide biosynthesis
*prsA_1*	*prsA_1*	*prsA_1*	5.2.1.8	Foldase protein PrsA	Protein secretion
*prsA_2*	*prsA_2*	*prsA_2*	5.2.1.8	Foldase protein PrsA	

**Table 7:** GO categories characterising the gene network for the L21 vs. L42 comparison: those present in L42 but absent in L21 (Figure 7A).

Colour	Term ID	Term Description	Observed Gene Count	False Discovery Rate
red	CL:3513	Mixed, incl. Oxo-acid-lyase activity, and Symport	8	1.44 × 10^−8^
blue	CL:3474	Mixed, incl. Glycerophospholipid metabolism, and Two-component system	10	3.22 × 10^−8^
lime-green	CL:3517	Oxo-acid-lyase activity, and Acyl carrier activity	5	7.48 × 10^−6^
yellow	CL:4045	Transposition, and DNA replication initiation	4	0.0027
magenta	CL:4057	DNA replication initiation, and Transposition	3	0.0082

**Table 8:** GO categories characterising the gene network for the L42 vs. L21 comparison: those present in L42 but absent in L21 (Figure 7B).

Colour	Term ID	Term Description	Observed Gene Count	False Discovery Rate
darkgreen	GOCC:0098533	ATPase dependent transmembrane transport complex	7	0.00018
red	CL:2853	P-type potassium transmembrane transporter activity	5	0.00019
lime-green	CL:2693	ABC transporters, and Peptide transport	10	0.0016
magenta	CL:2695	ABC transporters, and Nickel cation transmembrane transporter activity	9	0.0035
blue	CL:3353	Mixed, incl. Copper ion transport, and Ferric iron binding	4	0.0035
yellow	CL:3028	Mixed, incl. Entry into host, and Collagen binding	3	0.0183
cyan	GO:0055085	Transmembrane transport	15	0.0443

**Table 9:** GO categories characterising the gene network for the L42 vs. L51 comparison: those present in L42 but absent in L51 (Figure 8A).

Colour	Term ID	Term Description	Observed Gene Count	False Discovery Rate
blue	GO:0140097	Catalytic activity, acting on DNA	9	0.0352
lime-green	CL:2293	Mixed, incl. Polysaccharide biosynthetic process, and O-Antigen nucleotide sugar biosynthesis	6	0.0173
magenta	CL:2298	Polyketide sugar unit biosynthesis	3	0.035
yellow	CL:3028	Mixed, incl. Entry into host, and Collagen binding	3	0.035
darkgreen	CL:2759	Nickel cation transmembrane transporter activity, and Periplasmic space	3	0.035
cyan	CL:2296	Mixed, incl. Polyketide sugar unit biosynthesis, and Galactose metabolism	4	0.035
red	GO:0006259	DNA metabolic process	13	0.0426

**Table 10:** GO categories characterising the gene network for the L51 vs. L42 comparison: those present in L51 but absent in L42 (Figure 8B).

Colour	Term ID	Term Description	Observed Gene Count	False Discovery Rate
magenta	map00520	Amino sugar and nucleotide sugar metabolism	6	0.0019
red	CL:2607	Mixed, incl. O-Antigen nucleotide sugar biosynthesis, and Capsule organisation	7	1.45 × 10^−6^
blue	CL:2608	O-Antigen nucleotide sugar biosynthesis, and Galactose metabolic process	6	4.62 × 10^−6^
yellow	CL:2423	Mixed, incl. Starch and sucrose metabolism, and Carbohydrate metabolic process	8	0.0025

**Table 11:** GO categories characterising the gene network for the L51 vs. L21 comparison: those present in L51 but absent in L21 (Figure 9A).

Colour	Term ID	Term Description	Observed Gene Count	False Discovery Rate
cyan	GOCC:0031004	Potassium ion-transporting ATPase complex	3	0.0043
dark green	GO:0019829	ATPase-coupled cation transmembrane transporter activity	4	0.0058
red	CL:2267	P-type potassium transmembrane transporter activity, and Phosphorelay signal transduction system	5	9.57 × 10^−6^
blue	CL:2607	Mixed, incl. O-Antigen nucleotide sugar biosynthesis, and Capsule organisation	5	0.0014
lime-green	CL:2608	O-Antigen nucleotide sugar biosynthesis, and Galactose metabolic process	4	0.0059
yellow	CL:2601	Mixed, incl. Polysaccharide biosynthetic process, and Phosphotransferase activity, for other substituted phosphate groups	6	0.0059
magenta	CL:2610	O-Antigen nucleotide sugar biosynthesis, and Galactose metabolism	3	0.0226

**Table 12:** GO categories characterising the gene network for the L21 vs. L51 comparison: those present in L21 but absent in L51 (Figure 9B).

Colour	Term ID	Term Description	Observed Gene Count	False Discovery Rate
cyan	GOCC:0031004	Potassium ion-transporting ATPase complex	3	
dark green	GO:0019829	ATPase-coupled cation transmembrane transporter activity	4	0.0058
red	CL:2267	P-type potassium transmembrane transporter activity, and Phosphorelay signal transduction system	5	9.57 × 10^−6^
blue	CL:2607	Mixed, incl. O-Antigen nucleotide sugar biosynthesis, and Capsule organisation	5	0.0014
lime-green	CL:2608	O-Antigen nucleotide sugar biosynthesis, and Galactose metabolic process	4	0.0059
yellow	CL:2601	Mixed, incl. Polysaccharide biosynthetic process, and Phosphotransferase activity, for other substituted phosphate groups	6	0.0059
magenta	CL:2610	O-Antigen nucleotide sugar biosynthesis, and Galactose metabolism	3	0.0226

## Data Availability

The original contributions presented in this study are included in the article. Further inquiries can be directed to the corresponding author.

## Appendix A

**Table A1:** Products of the genes that differentiate strains L42 and L21.

N	L42	L21
1.	ABC transporter ATP-binding protein YtrB	[Citrate [pro-3S]-lyase] ligase
2.	Bacteriocin lactococcin B	2-(5″-triphosphoribosyl)-3′-dephosphocoenzyme-A synthase
3.	Cadmium resistance transcriptional regulatory protein CadC	2-C-methyl-D-erythritol 4-phosphate cytidylyltransferase
4.	Cadmium-transporting ATPase	Apo-citrate lyase phosphoribosyl-dephospho-CoA transferase
5.	Curved DNA-binding protein	Bis(5′-nucleosyl)-tetraphosphatase, symmetrical
6.	General stress protein 18	Citrate lyase acyl carrier protein
7.	Glycosyltransferase GlyD	Citrate lyase alpha chain
8.	Group II intron-encoded protein LtrA	Citrate lyase subunit beta
9.	Homoserine O-succinyltransferase	Citrate/malate transporter
10.	HTH-type transcriptional repressor YtrA	D-alanyl-D-alanine carboxypeptidase DacA
11.	IS21 family transposase IS712	Galactose/methyl galactoside import ATP-binding protein MglA
12.	IS3 family transposase IS1163	GDP-L-fucose synthase
13.	IS3 family transposase IS981	GDP-mannose 4,6-dehydratase
14.	IS3 family transposase IS-LL6	Glucitol operon repressor
15.	IS3 family transposase ISSag5	HTH-type transcriptional regulator Hpr
16.	IS3 family transposase ISWci2	Intermembrane phospholipid transport system ATP-binding protein MlaF
17.	IS4 family transposase IS1675	IS256 family transposase IS1310
18.	K(+)/H(+) antiporter NhaP2	IS256 family transposase IS905
19.	Multidrug resistance protein 3	IS256 family transposase ISEfm2
20.	N-acetyl-alpha-D-glucosaminyl L-malate synthase	IS6 family transposase ISS1W
21.	NADP-dependent 3-hydroxy acid dehydrogenase YdfG	IS6 family transposase ISTeha2
22.	Nitrogen fixation regulation protein FixK	IS982 family transposase ISLll1
23.	Oligopeptide-binding protein AppA	ISL3 family transposase ISSpn3
24.	Potassium-transporting ATPase KdpC subunit	L-threonine 3-dehydrogenase
25.	Potassium-transporting ATPase potassium-binding subunit	NAD-dependent malic enzyme
26.	putative ABC transporter ATP-binding protein YheS	Oxidoreductase YdhF
27.	putative ATP-dependent Clp protease ATP-binding subunit	Peptide transporter CstA
28.	putative HTH-type transcriptional regulator YdjF	PTS system oligo-beta-mannoside-specific EIIC component
29.	putative MFS-type transporter YfcJ	putative acyl-CoA ligase YhfT
30.	Putative mycofactocin radical SAM maturase MftC	putative SapB synthase
31.	putative oxidoreductase YghA	Pyrrolidone-carboxylate peptidase
32.	putative protein YqbD	Sorbitol operon regulator
33.	Pyruvate/proton symporter BtsT	Spore coat protein SA
34.	Replication initiation protein	Staphylopine export protein
35.	Riboflavin biosynthesis protein	Transposase from transposon Tn916
36.	Sensor protein KdpD	Tyrosine recombinase XerD
37.	Sensory transduction protein regX3	UDP-galactopyranose mutase
38.	Serine/threonine-protein kinase PknD	
39.	SkfA peptide export ATP-binding protein SkfE	
40.	Teichuronic acid biosynthesis protein TuaB	
41.	Trans-aconitate 2-methyltransferase	
42.	Transcriptional regulatory protein KdpE	
43.	UDP-N-acetylglucosamine 4-epimerase	
44.	Xylose import ATP-binding protein XylG	

**Table A2:** Products of the genes that differentiate strains L42 and L51.

N	L42	L51
1.	ABC transporter ATP-binding protein YtrB	[LysW]-aminoadipate semialdehyde transaminase
2.	ADP-L-glycero-D-manno-heptose-6-epimerase	1-(5-phosphoribosyl)-5-[(5-phosphoribosylamino) methylideneamino] imidazole-4-carboxamide isomerase
3.	Branched-chain amino acid transport system 2 carrier protein	2,5-diketo-D-gluconic acid reductase A
4.	Cadmium resistance transcriptional regulatory protein CadC	2-C-methyl-D-erythritol 4-phosphate cytidylyltransferase
5.	Curved DNA-binding protein	Aconitate hydratase A
6.	DNA topoisomerase 3	Arsenical pump-driving ATPase
7.	DNA-invertase hin	Arsenical resistance operon trans-acting repressor ArsD
8.	dTDP-4-dehydrorhamnose 3,5-epimerase	Arsenical resistance protein Acr3
9.	General stress protein 18	D-alanyl-D-alanine carboxypeptidase DacA
10.	Glycosyltransferase GlyD	Galactose/methyl galactoside import ATP-binding protein MglA
11.	Homoserine O-succinyltransferase	Glucitol operon repressor
12.	HTH-type transcriptional repressor YtrA	HTH-type transcriptional regulator Hpr
13.	IS21 family transposase IS712	Inner membrane protein YagU
14.	IS3 family transposase IS1163	IS6 family transposase ISTeha2
15.	IS3 family transposase ISLla3	L-threonine 3-dehydrogenase
16.	IS3 family transposase ISWci2	Modification methylase FokI
17.	IS4 family transposase IS1675	Oxidoreductase YdhF
18.	IS5 family transposase IS1194	PTS system oligo-beta-mannoside-specific EIIC component
19.	IS982 family transposase ISLla2	putative acyl--CoA ligase YhfT
20.	K(+)/H(+) antiporter NhaP2	Putative O-antigen transporter
21.	N-acetyl-alpha-D-glucosaminyl L-malate synthase	Staphylopine export protein
22.	NADP-dependent 3-hydroxy acid dehydrogenase YdfG	Type-2 restriction enzyme BsuMI component YdiS
23.	Oligoendopeptidase F, plasmid	UDP-galactopyranose mutase
24.	Oligopeptide-binding protein AppA	UDP-glucose 4-epimerase
25.	Phosphoribosyl isomerase A	UDP-glucose 6-dehydrogenase
26.	putative ABC transporter ATP-binding protein YheS	[LysW]-aminoadipate semialdehyde transaminase
27.	putative HTH-type transcriptional regulator YdjF	
28.	putative MFS-type transporter YfcJ	
29.	Putative mycofactocin radical SAM maturase MftC	
30.	putative oxidoreductase YghA	
31.	putative protein YjdJ	
32.	putative protein YqbD	
33.	Pyruvate/proton symporter BtsT	
34.	Replication initiation protein	
35.	Riboflavin biosynthesis protein	
36.	Sensory transduction protein regX3	
37.	Serine/threonine-protein kinase PknD	
38.	SkfA peptide export ATP-binding protein SkfE	
39.	Teichuronic acid biosynthesis protein TuaB	
40.	Type-1 restriction enzyme R protein	
41.	UDP-N-acetylglucosamine 4-epimerase	
42.	Xylose import ATP-binding protein XylG	
43.	ABC transporter ATP-binding protein YtrB	
44.	ADP-L-glycero-D-manno-heptose-6-epimerase	

**Table A3:** Products of the genes that differentiate strains L51 and L21.

N	L51	L21
1.	[LysW]-aminoadipate semialdehyde transaminase	[Citrate [pro-3S]-lyase] ligase
2.	1-(5-phosphoribosyl)-5-[(5-phosphoribosylamino)methylideneamino] imidazole-4-carboxamide isomerase	2-(5″-triphosphoribosyl)-3′-dephosphocoenzyme-A synthase
3.	2,5-diketo-D-gluconic acid reductase A	ADP-L-glycero-D-manno-heptose-6-epimerase
4.	Aconitate hydratase A	Apo-citrate lyase phosphoribosyl-dephospho-CoA transferase
5.	Arsenical pump-driving ATPase	Bis(5′-nucleosyl)-tetraphosphatase, symmetrical
6.	Arsenical resistance operon trans-acting repressor ArsD	Branched-chain amino acid transport system 2 carrier protein
7.	Arsenical resistance protein Acr3	Citrate lyase acyl carrier protein
8.	Bacteriocin lactococcin B	Citrate lyase alpha chain
9.	Cadmium-transporting ATPase	Citrate lyase subunit beta
10.	Group II intron-encoded protein LtrA	Citrate/malate transporter
11.	Inner membrane protein YagU	DNA topoisomerase 3
12.	IS3 family transposase IS981	DNA-invertase hin
13.	IS3 family transposase IS-LL6	dTDP-4-dehydrorhamnose 3,5-epimerase
14.	IS3 family transposase ISSag5	GDP-L-fucose synthase
15.	Modification methylase FokI	GDP-mannose 4,6-dehydratase
16.	Multidrug resistance protein 3	Intermembrane phospholipid transport system ATP-binding protein MlaF
17.	Nitrogen fixation regulation protein FixK	IS256 family transposase IS1310
18.	Potassium-transporting ATPase KdpC subunit	IS256 family transposase IS905
19.	Potassium-transporting ATPase potassium-binding subunit	IS256 family transposase ISEfm2
20.	putative ATP-dependent Clp protease ATP-binding subunit	IS3 family transposase ISLla3
21.	Putative O-antigen transporter	IS5 family transposase IS1194
22.	Sensor protein KdpD	IS6 family transposase ISS1W
23.	Trans-aconitate 2-methyltransferase	IS982 family transposase ISLla2
24.	Transcriptional regulatory protein KdpE	IS982 family transposase ISLll1
25.	Type-2 restriction enzyme BsuMI component YdiS	ISL3 family transposase ISSpn3
26.	UDP-glucose 4-epimerase	NAD-dependent malic enzyme
27.	UDP-glucose 6-dehydrogenase	Oligoendopeptidase F, plasmid
28.	[LysW]-aminoadipate semialdehyde transaminase	Peptide transporter CstA
29.		Phosphoribosyl isomerase A
30.		putative protein YjdJ
31.		putative SapB synthase
32.		Pyrrolidone-carboxylate peptidase
33.		Sorbitol operon regulator
34.		Spore coat protein SA
35.		Transposase from transposon Tn916
36.		Type-1 restriction enzyme R protein
37.		Tyrosine recombinase XerD
